# Supplementary material for: The interactome of intact mitochondria by cross-linking mass spectrometry provides evidence for coexisting respiratory supercomplexes
Source: Mol Cell Proteomics. 2017 Dec 8;17(2):216–32. doi: 10.1074/mcp.RA117.000470 (PMC5795388; doi:10.1074/mcp.RA117.000470)
Supplement: Supplemental Data [file supp_RA117.000470_133922_0_supp_23978_fzffwf.zip › spectra_annotation/mito_spectra_annotation/463-1-10-1-18-1.pdf]

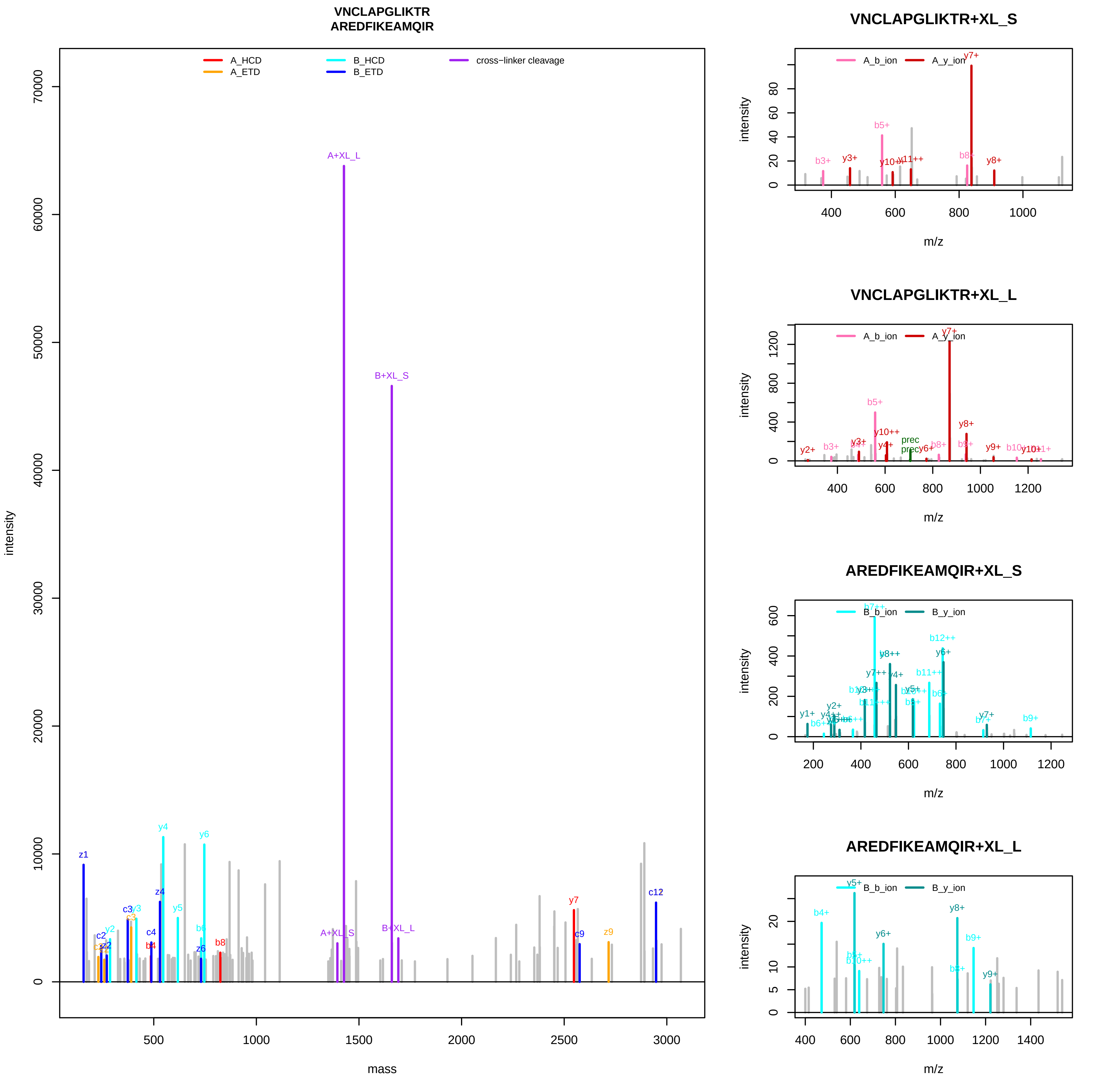
VNCLAPGLIKTR
AREDFIKEAMQIR
A_HCD
A_ETD
B_HCD
B_ETD
cross−linker cleavage
intensity
mass
A+XL_L
B+XL_S
A+XL_S
B+XL_L
z1
y4
y6
z4
c3
y5
c4
b4
b8
y7
c9
z9
c12
z6
VNCLAPGLIKTR+XL_S
A_b_ion
A_y_ion
y7+
b5+
b3+
y3+
b8+
y8+
m/z
VNCLAPGLIKTR+XL_L
y8+
y2+
y4+
y9+
prec
AREDFIKEAMQIR+XL_S
B_b_ion
B_y_ion
b12++
y6+
y8++
y7++
y4+
b11++
y1+
y2+
y7+
b9+
AREDFIKEAMQIR+XL_L
y5+
b4+
y8+
y6+
b9+
y9+
